# Randomized target engagement trial of a dissonance-based transdiagnostic eating disorder treatment versus transdiagnostic interpersonal psychotherapy

**DOI:** 10.1017/S0033291725102341

**Published:** 2025-11-06

**Authors:** Eric Stice, Sonja Yokum, Paul Rohde, Cara Bohon, Heather Shaw

**Affiliations:** 1 Department of Psychiatry and Behavioral Sciences, Stanford University, Stanford, CA, USA; 2 Oregon Research Institute, Springfield, OR, USA

**Keywords:** cognitive dissonance, eating disorders, fMRI, interpersonal psychotherapy, transdiagnostic, treatment

## Abstract

**Background:**

Test whether a dissonance-based transdiagnostic eating disorder treatment, *body project treatment* (BPT), produces greater reduction in brain reward region response to the thin ideal and behaviors used to pursue this ideal and eating disorder symptoms, and higher abstinence from eating disorder behaviors and remittance from eating disorder diagnoses than a matched transdiagnostic *interpersonal psychotherapy* (IPT).

**Methods:**

Women with various eating disorders (*N* = 83) were randomized to 8-week group-implemented BPT or IPT and completed functional magnetic resonance imaging (fMRI) at pretest and posttest, and surveys and masked diagnostic interviews at pretest, posttest, and 6-month follow-up.

**Results:**

BPT versus IPT participants showed significantly greater reductions in mid cingulate cortex response to thin models, anterior cingulate cortex response to eating disorder behavior words, eating disorder symptoms (*d* = 0.54), and body dissatisfaction (*d* = 0.57), and marginally greater reductions in psychosocial impairment (*d* = 0.39) at posttest, as well as significantly greater reductions in body dissatisfaction (*d* = 0.68) and psychosocial impairment (*d* = 0.63), and marginally greater reductions in eating disorder symptoms (*d* = 0.53) at 6-month follow-up. At posttest, BPT versus IPT participants showed significantly greater abstinence from binge eating and purging (48% versus 23%, respectively) but did not differ on remittance from eating disorder diagnoses (52% versus 44%, respectively).

**Conclusions:**

Results provide further evidence of target engagement for BPT and suggest that it is more effective than IPT in treating a range of eating disorders.

## Public Health Impact Statement

This study provides evidence that a novel dissonance-based transdiagnostic eating disorder treatment, *Body Project Treatment (BPT)*, produced greater reductions in eating disorder symptoms, abstinence from binge eating and purging behaviors, body dissatisfaction, and psychosocial impairment than group-delivered transdiagnostic *Interpersonal Psychotherapy.* Further, brain imaging data provided evidence that *BPT* reduced valuation and salience of the thin ideal. This dissonance-based treatment is one of the only eating disorder treatments to produce significantly greater clinical benefit than an active alternative eating disorder treatment.

Eating disorders, which include threshold and subthreshold anorexia nervosa (AN), bulimia nervosa (BN), and binge eating disorder (BED), as well as purging disorder (PD) are marked by emotional distress, psychosocial impairment, relapse, and mortality (Allen, Byrne, Oddy, & Crosby, 2013; Stice, Marti, & Rohde, 2013). However, 80% of affected US individuals do not receive treatment (Penwell, Bedard, Eyre, & Levinson, 2024), largely because most evidence-based treatments involve at least 20 individual sessions. Treatment cost or lack of insurance coverage is the most frequently cited reason for not using mental health services (SAMSA, 2015). This service shortfall has prompted interest in briefer transdiagnostic eating disorder treatments that would be easier to implement.

Among transdiagnostic treatments, *Body Project Treatment* (BPT; Stice et al., 2015) is the most cost-effective because it is delivered in 8 1-hour group sessions rather than 20 individual sessions for other transdiagnostic treatments, such as *Interpersonal Psychotherapy* (IPT), *Enhanced Cognitive Behavior Therapy* (CBT-E), and *Integrative Cognitive-Affective Therapy* (ICAT; Fairburn et al., 2015; Wonderlich et al., 2014). In BPT, women with eating disorders appropriate for outpatient care collectively discuss the negative effects of pursuing the thin ideal and eating disorder behaviors, which putatively creates cognitive dissonance that prompts them to reduce valuing the thin ideal and behaviors used to pursue this ideal because people are motivated to align their cognitions with their publicly displayed behaviors (Rohde, Stice, & Marti, 2015). Reducing valuation of the thin ideal is a key intervention target because pursuit of the thin ideal predicted future onset of eating disorders (Allen et al., 2014; Dakanalis et al., 2017; Stice, Desjardins, Rohde, & Shaw, 2021b) and future persistence of eating disorder symptoms (Bohon, Stice, & Burton, 2009; Dakanalis et al., 2017; Fairburn et al., 2003; Stice, Bohon, Gau, & Rohde, 2021a). Further, elevated reward region response to the thin ideal predicted future onset of binge eating and compensatory weight control behaviors (Stice, Yokum, Gau, & Shaw, 2025) and future eating disorder symptom persistence (Stice et al., 2023). Theoretically, pursuing the thin ideal increases risk for fasting and other unhealthy weight control behaviors, which in turn increase risk for binge eating. We also expected that discussing the costs of binge eating would reduce the valuation of binge foods. Binge eating predicted future eating disorder onset (Stice, Desjardins, et al., 2021b; Yamamiya, Desjardins, & Stice, 2023) and eating disorder symptom persistence (Stice, Bohon, et al., 2021a), theoretically because binge eating increases risk for unhealthy weight control behaviors. However, reward region response to binge foods did not predict future eating disorder symptom persistence (Stice & Yokum, 2023).

BPT versus usual-care control participants showed greater reductions in pursuing the thin ideal, body dissatisfaction, negative affect, and eating disorder symptoms through 2-month follow-up in an initial trial (*N* = 72; Stice, Rohde, Butryn, et al., 2015). BPT participants showed lower body dissatisfaction and negative affect at posttest and 6-month follow-up, and social impairment at 6-month follow-up than participants randomized to a supportive mindfulness group treatment in a second trial (*N* = 84; Stice, Rohde, Shaw, & Gau, 2019). By 6-month follow-up, more BPT participants no longer met diagnostic criteria for an eating disorder than supportive mindfulness participants (77% vs 60%), though there was no difference in eating disorder symptom change. BPT participants showed non-significantly higher abstinence from binge eating and purging behaviors in the past month than supportive mindfulness participants (55% versus 39%) by 6-month follow-up.

Because it is valuable to experimentally investigate whether treatments alter the intervention target and a change in the target correlates with symptom reduction, as this approach can identify factors that maintain psychopathology, a third trial used functional magnetic resonance imaging (fMRI) to evaluate target engagement (*N* = 138; Stice et al., 2023). Previous trials have used fMRI to evaluate target engagement for other prevention programs and treatments (Borgers et al., 2025; Norman et al., 2021; Stice et al., 2015b, 2017; Stice, Yokum, & Waters, 2015). BPT versus waitlist controls showed greater reductions in responsivity of brain regions implicated in reward valuation (caudate) and attentional motivation (precuneus) to thin models, as well as greater reductions in responsivity of brain regions implicated in reward valuation (ventrolateral prefrontal cortex [vlPFC]) and food craving (hippocampus) to high-calorie binge foods. BPT versus control participants also showed greater reductions in eating disorder symptoms, abstinence from binge eating and purging, palatability ratings, and monetary value ratings of high-calorie binge foods, pursuit of the thin ideal, and greater increases in attractiveness ratings of average weight models (Stice, Yokum et al., 2019, Stice et al., 2023).

In a fourth trial, participants randomized to BPT versus *Interpersonal Psychotherapy* (IPT) that was matched in terms of group-delivery and intervention duration showed greater reductions in eating disorder symptoms, pursuit of the thin ideal, anxiety symptoms, and social impairment through 6-month follow-up (*N* = 73; Stice, Rohde et al., 2023). We selected IPT as the alternative intervention because it is an evidence-based treatment that focuses on a distinct intervention target (i.e., interpersonal difficulties). Group-delivered IPT has produced greater reductions in eating disorder symptoms than waitlist controls but not cognitive-behavioral therapy (Wilfley et al., 1993, 2002). By end of treatment, participants randomized to BPT versus IPT showed non-significantly higher abstinence from binge eating and purging (49% versus 40%, respectively) and remittance from eating disorder diagnoses (54% versus 40%, respectively).

Unfortunately, because of COVID-related social distancing policies, we were unable to evaluate target engagement with fMRI in the fourth trial that compared BPT to IPT. Accordingly, the present trial tested the hypothesis that participants treated with BPT versus IPT would show greater reductions in responsivity of reward valuation regions in response to thin models and high-calorie binge foods. Although we hypothesized differential changes in reward region responsivity, we used a whole-brain approach to allow detection of broader neural responses. We also hypothesized that participants randomized to BPT versus IPT would show greater reductions in eating disorder symptoms (primary outcome) and pursuit of the thin ideal. These hypotheses are based on evidence that valuing the thin ideal and behaviors used to pursue this ideal predicted future persistence of eating disorder symptoms, whereas psychosocial functioning impairment did not (Bohon et al., 2009; Dakanalis et al., 2017; Fairburn et al., 2003; Stice, Bohon, et al., 2021a) and evidence that BPT produced a higher abstinence from binge eating and purging (48% on average; Stice et al., 2019; Stice, Yokum et al., 2019; Stice, Rohde et al., 2023; Stice et al., 2023) than IPT (38% on average; Fairburn et al., 2015; Stice, Rohde et al., 2023). Further, we hypothesized that BPT would produce higher abstinence from binge eating and purging, and higher remittance from eating disorders than IPT.

## Methods

### Participants and procedure

**Figure 1.**
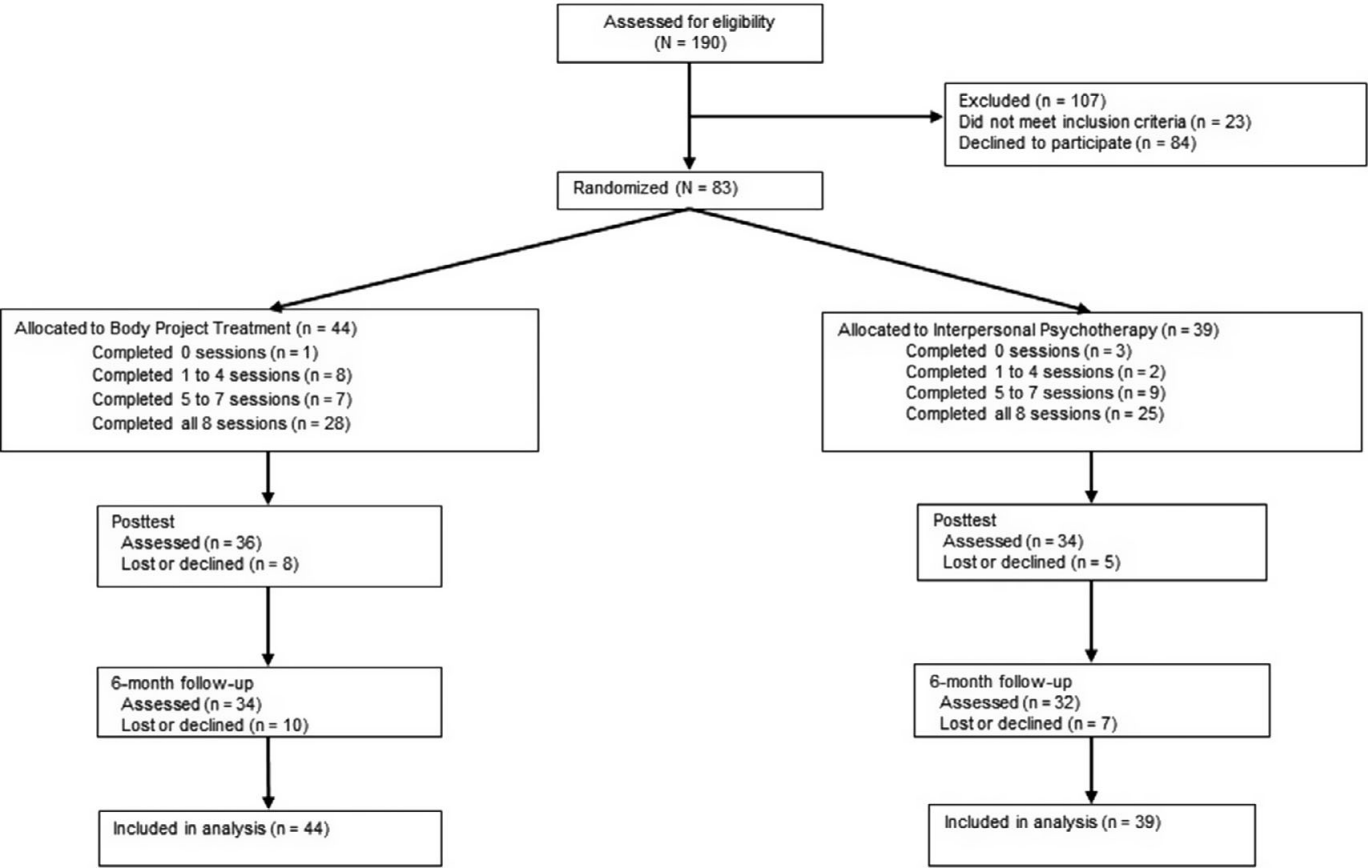
Participant flow throughout the study.

We recruited 83 women (*M* age = 23.5 ± 5.0) in California and Oregon. Web postings, flyers, and mailings invited women to participate in a trial comparing two eating disorder treatments. We also asked local clinics that treat eating disorders to refer participants. Informed consent was obtained for this institutional review board-approved trial. Inclusion criteria were having a DSM-5 eating disorder appropriate for outpatient care and being at least 18 years of age. Women with a body mass index (BMI; kg/m^2^) below 17 were excluded because they were not appropriate for outpatient treatment without medical monitoring. Current suicidal ideation and substance abuse were also exclusion criteria. At baseline, 13% participants met criteria for threshold/subthreshold AN, 46% for threshold/subthreshold BN, 31% for threshold/subthreshold BED, and 10% for PD. See Table 1 for demographics of the sample. Participants were randomized to BPT (*N* = 44) or IPT (*N* = 39) using a random number table by the project coordinator. Assessors were masked to the condition. Participants completed fMRI scans at pretest and posttest, and surveys and diagnostic interviews at pretest, posttest, and at 6-month follow-up (see Figure 1 for the flow chart).

**Table 1.** Demographic variables by study condition

	IPT		BPT		Test statistic	*p*
Age [Mean, (SD)]	24.82	(6.07)	22.14	(3.44)	*t*(81) = 2.62	.011
Hispanic (%)		20.5		18.6	*χ* ^2^(1,82) = 0.05	.838
Race (%)					*χ* ^2^(5,82) = 11.72	.039
Native American		2.6		2.3		
Asian		25.7		34.9		
White		41.0		55.8		
Native Hawaiian/Pacific Islander		0.0		2.3		
Black		17.9		0.0		
Multiracial		12.8		4.7		
Maximum parental education (%)					*χ* ^2^(5,82) = 2.60	.762
Grade school graduate		0.0		2.3		
Some high school		7.7		4.7		
High school graduate		7.7		9.3		
Some college		5.1		2.3		
College graduate		23.1		32.6		
Advanced degree		56.4		48.8		
Binge episodes past month (Mean/SD)		6.38 (7.38)		5.16 (5.14)	*t*(81) = 0.89	.378
Purge episodes past month (Mean/SD)		2.03 (4.70)		2.80 (4.60)	*t*(81) = −0.75	.454

*Note*: BPT, *Body Project Treatment*; IPT, Interpersonal Psychotherapy; *p*, *p*-value; SD, standard deviation.

#### Body project treatment (BPT)

BPT consisted of 8 weekly 1-hour in-person group sessions with 6–8 participants led by a therapist. During sessions, participants completed written, verbal, and behavioral exercises, including defining the thin ideal, discussing costs of pursuing this ideal and eating disorder symptoms, role-plays in which participants dissuade facilitators from pursuing the thin ideal and engaging in eating disorder symptoms, motivational exercises (e.g., discussing the importance of addressing their eating disorder), and shared their home exercises. Home exercises included writing letters (e.g., to their eating disorder), motivational exercises (e.g., writing about the importance of improving body acceptance), a mirror body appreciation exercise, generating lists of “body activism” behaviors women can do to resist the thin ideal, reducing “linchpin” eating disorder symptoms (including eating 3 healthy meals daily to reduce fasting), and tracking binge episodes and compensatory behaviors. See https://sticebodyprojectsupport.weebly.com for the intervention manual.

#### Interpersonal psychotherapy (IPT)

IPT was initially developed as an individual treatment for depression (Markowitz & Weissman, 2012) but has since been adapted for several populations and formats, including group-delivered eating disorder treatment (Stice, Rohde et al., 2023; Wilfley et al., 1993, 2002), group-delivered eating disorder prevention program (Tanofsky-Kraff et al., 2007, 2010; Tanofsky-Kraff et al., 2014), and individually delivered eating disorder treatment (Fairburn et al., 2015; Fairburn et al., 1993). We used the transdiagnostic group-delivered version of IPT that we evaluated previously (Stice, Rohde et al., 2023) that matched BPT in duration and modality. The 8-session in-person group retained key components of IPT including a group interpersonal inventory, identifying problem areas, focusing on the present, skill development and home practice, and relationship building exercises, based on prior manuals (Mufson, Dorta, Moreau, & Weissman, 2011; Wilfley et al., 2000; Wilson, Wilfley, Agras, & Bryson, 2010; World Health Organization & Columbia University, 2016; Young, Mufson, & Schueler, 2016). Sessions focus on current interpersonal challenges, the links between interpersonal challenges and eating disorder symptoms, and resolving interpersonal challenges. In the initial treatment phase (sessions 1–2), the goal is to introduce IPT and group members, introduce problem areas and set goals, and discuss how the group works. In the middle phase (sessions 3–7), the therapist guides participants through learning and practicing skills to improve interpersonal challenges using role-plays and peer support. In the termination phase (session 8), skills and achievements are reviewed.

Facilitators in both conditions were clinical psychology graduate students. Training involved reading the manual and attending an 8-hr workshop to learn the intervention rationale, role-play session delivery, and discussing process issues. Drs. Stice and Rohde provided BPT training, and Drs. Bohon and Rohde provided IPT training. Sessions were video-recorded and reviewed by Drs. Rohde, Shaw, and Bohon, who rated session adherence and therapeutic competence using rating scales and provided emailed supervision.

### Measures

#### fMRI Paradigms

Participants completed a thin model exposure paradigm, a high-calorie binge food exposure paradigm, and an eating disorder behavior word paradigm. During the thin model exposure paradigm, participants viewed images of thin and average-weight models (20 events each) and were asked to think about the attractiveness of each model. During the high-calorie binge food exposure paradigm, participants viewed images of high-calorie binge foods (e.g., chocolate cake) and low-calorie foods (fruits and vegetables) (20 events each) and were asked to think about how much they wanted to eat each food. These fMRI paradigms have shown predictive validity for future onset and persistence of eating disorder symptoms (Stice et al., 2025; Stice & Yokum, 2023) and sensitivity to detecting intervention effects (Stice, Yokum, Burger et al., 2015; Stice, Yokum, Rohde, et al., 2023; Stice, Yokum, Veling et al., 2017; Stice, Yokum, Waters, 2015). During the eating disorder behavior word paradigm developed for this trial, participants viewed words describing eating disorder behaviors (e.g., binge, purge) versus words describing neutral behaviors (e.g., talk, drive; 20 events each) and were asked to think about how they feel about performing each action. In all 3 paradigms, events were presented for 5 seconds in a randomized order, followed by a 4–8 second jittered fixation cross. Order of paradigms and order of presentation of the pictures and words were randomized across participants.

#### Eating disorder symptoms

The *Eating Disorder Diagnostic Interview* (EDDI; Stice et al., 2013) assessed symptoms for DSM-5 eating disorders (American Psychiatric Association, 2013). It assessed symptoms on a month-to-month basis in the past 3 months at pretest and since the last assessment at posttest and 6-month follow-up. We calculated a continuous symptom composite, which reflected symptoms in the past month. This composite has shown internal consistency (*α* = .92), inter-rater agreement (ICC *r* = .93), 1-week test–retest reliability (ICC *r* = .95), and sensitivity to detecting effects of eating disorder treatments (Stice et al., 2023; Stice, Desjardins, et al., 2021b); *α* = .79 at pretest. We also examined abstinence, defined as no binge eating or purging behaviors in the past month, as well as remittance of eating disorder diagnoses at posttest and 6-month follow-up. EDDI eating disorder diagnoses have shown 1-week test–retest reliability (κ = .79), inter-rater agreement (κ = .75), and sensitivity to detecting intervention effects (Stice et al., 2019; Stice, Desjardins, et al., 2021b).

#### Pursuit of the thin ideal

The 8-item Thin-Ideal Internalization Scale assessed pursuing the thin ideal (Stice, Desjardins, et al., 2021b). This self-report scale has shown 2-week test–retest reliability (*r* = .80), predictive validity for onset of BN, BED, and PD, and sensitivity to detecting intervention effects (Stice, Desjardins, et al., 2021b; Stice, Rohde, Butryn, et al., 2015); *α* = .72 at pretest.

#### *Depression* symptoms

The Patient Health Questionnaire (PHQ-9; Kroenke, Spitzer, & Williams, 2001) measured depressive symptoms over the last 2 weeks with nine items. This self-report scale has shown internal consistency (*α* = .89), 2-week test–retest reliability (*r* = .73), and convergent validity (e.g., Sun et al., 2020); *α* = .88 at pretest.

#### *Anxiety* symptoms

The General Anxiety Disorder-7 (GAD-7; Spitzer, Kroenke, Williams, & Lowe, 2006) measured anxiety symptoms over the last 2 weeks using seven items. This self-report scale has shown internal consistency (*α* > .82) and convergent validity (Johnson, Ulvenes, Øktedalen, & Hoffart, 2019); *α* = .88 at pretest.

#### *Psychosocial* Impairment

Impairment in psychosocial functioning in the family, peer group, romantic, and work domains was measured with 17 items from the Social Adjustment Scale (Weissman & Bothwell, 1976). This self-report scale has shown internal consistency (*α* = .77), 1-week test–retest reliability (*r* = .83), predictive validity for future AN, BN, BED, and PD onset (Stice, Desjardins, et al., 2021b); *α* = .84 at pretest.

### Statistical methods

#### *Preliminary* analyses

We examined the distribution of outcomes and normalized data when needed to minimize the impact of outliers and reduce residual heterogeneity. Comparisons between conditions were made for pretest values of outcomes and demographics to assess group equivalency. We compared participants who completed all assessments and those who did not on condition, pretest values of outcomes, and demographics.

#### *MRI acquisition and fMRI data* preprocessing

MRI data were acquired on a Siemens Skyra 3 T MRI scanner (Oregon; *N* = 17) and a GE Discovery MR750 scanner (California; *N* = 66). Neuroimaging data were preprocessed and analyzed using the Statistical Parametric Mapping (SPM12) in Matlab (Mathworks, Inc., Natick, MA). Head motion ≥3 mm or degrees in any direction was our *a priori* exclusion criteria. At pretest, one participant showed excessive head motion during the thin model paradigm and eating disorder behavior word paradigm output files were not recorded for two participants. At posttest, one participant showed excessive head motion during all three paradigms and one participant showed excessive head motion during the thin model paradigm. fMRI data of these participants were excluded. Complete fMRI data were available for 70 participants for the high-calorie binge food paradigm and eating disorder behavior word paradigm, and for 69 participants for the thin model paradigm. See Supplementary Material for greater details.

#### *fMRI data* analysis

We conducted two groups (BPT versus IPT) × 2 Time (pretest versus posttest) repeated-measures ANOVAs to examine intervention effects on change in neural response to the fMRI paradigms. Scan site was a covariate for all models. BMI was a covariate for the thin model and high-calorie food paradigms. Hours since last food intake was also a covariate for the latter paradigm. Whole-brain analyses were conducted. To control for multiple comparisons, we applied a voxel-wise threshold of p < 0.001 (uncorrected) and a cluster-level threshold of *p* < 0.05 (corrected) using random field theory in SPM12. fMRI effect sizes (*r*) were derived from the *Z*-values (*Z*/√*N*).

#### *Continuous and dichotomous* outcomes

For continuous and dichotomous outcomes (below), we imputed 50 complete datasets with SPSS and included baseline demographic characteristics and outcome variables at each assessment in the imputation model (Graham, 2009) to allow intent-to-treat analyses. Parameter estimates from the 50 imputed datasets were combined. Change in the continuous outcomes were assessed using one-way and mixed model analyses of variance. The models included fixed effects of time, condition (0 = IPT, 1 = BPT) and condition × time interaction. Cohen’s *d* (Cohen, 1988) was used to estimate effect size. We evaluated group differences in abstinence (0 = not abstinent; 1 = abstinent) and diagnostic remission (0 = no remission; 1 = remission). Logistic regression models, estimated in SPSS, included the posttest and 6-month rates as outcomes with condition and pretest rates as a covariate.

## Results

### Preliminary analyses

Eating disorder symptoms at posttest and 6-month follow-up were positively skewed. Winsorizing, wherein scores more than two standard deviations (SD) above the mean are replaced with less extreme values (scores at 2 SD), was used to make analyses more robust to outliers and satisfy the assumption of normal distributions.

BPT and IPT conditions significantly differed on age and percent Black, but did not differ on other pretest values (Table 1). All analyses controlled for pretest age and percent Black in the groups. Table 2 provides means and *SD* for continuous outcomes by group. Retention was 84% at posttest and 80% at 6-month follow-up. Attrition was not significantly related to condition, demographics, or pretest outcomes.

**Table 2.** Descriptive statistics for outcomes by condition

Group	Pretest		Posttest		6-Month	
	Mean	SD	Mean	SD	Mean	SD
Eating disorder symptoms						
*IPT*	33.69	18.01	26.53	19.94	18.01	13.70
*BPT*	36.75	19.69	20.58	15.10	17.26	15.30
Pursuit of the thin ideal						
*IPT*	3.77	0.48	3.49	0.57	3.53	0.61
*BPT*	3.76	0.46	3.58	0.56	3.52	0.40
Body dissatisfaction						
*IPT*	3.82	0.71	3.77	0.75	3.68	0.78
*BPT*	3.96	0.64	3.49	0.62	3.34	0.76
Anxiety symptoms						
*IPT*	1.29	0.78	1.50	1.08	1.09	0.65
*BPT*	1.34	0.69	1.18	0.77	1.09	0.67
Depressive symptoms						
*IPT*	1.18	0.65	1.08	0.57	0.97	0.50
*BPT*	1.24	0.64	0.95	0.57	0.89	0.61
Psychosocial impairment						
*IPT*	2.11	0.60	2.12	0.53	2.11	0.51
*BPT*	2.20	0.49	2.06	0.41	1.89	0.53

*Note*: Winsorized values of eating disorder symptoms reported for posttest and 6-month follow-up. BPT, *Body Project Treatment*; IPT, Interpersonal Psychotherapy; SD, standard deviation.

### Attendance and home exercise completion

Seventy-two participants attended ≥7 sessions (87%) and 70% completed ≥6 of the home exercises. BPT (*M* = 6.2, *SD* = 2.5) and IPT participants (*M* = 6.9, *SD* = 2.3) did not differ in average attendance (*p* = .154) or home exercise completion (BPT *M* = 5.3, *SD* = 2.7 versus IPT *M* = 5.9, *SD* = 2.0; *p* = .33).

### Intervention effects on neural response to thin models, high-calorie foods, and eating disorder behavior words

**Figure 2.**
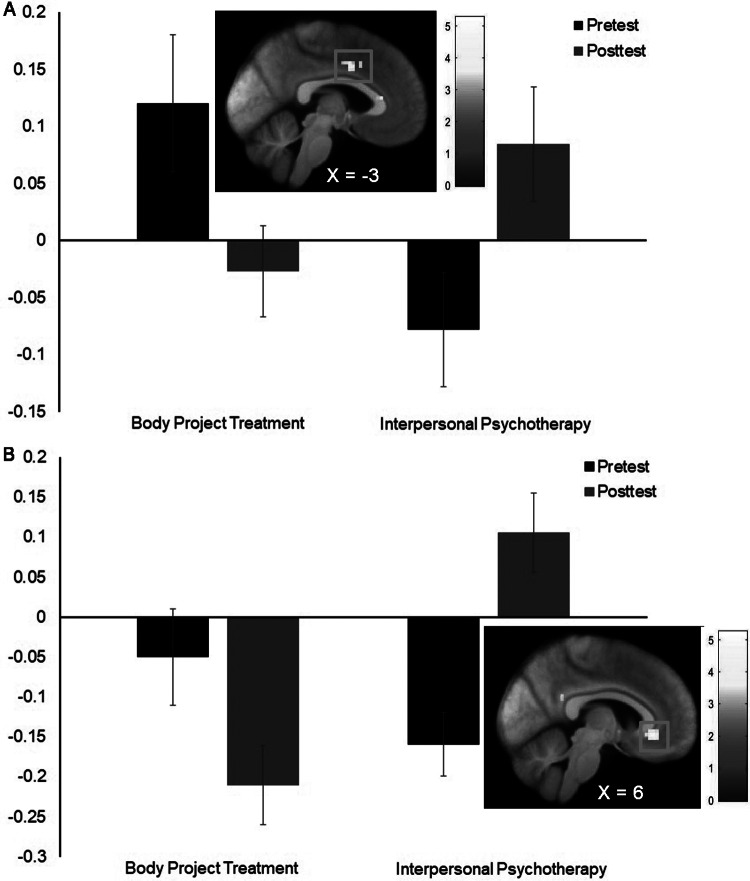
Greater pre- to post BOLD response decreases in (a) middle cingulate cortex (MCC MNI coordinates: −3, 2, 38, Z = 3.96, k = 34) in response to the thin model > average-weight model contrast and (b) anterior cingulate cortex (ACC MNI coordinates: 6, 32, −7, Z = 4.99, k = 53) in response to the eating disorder behavior words > neutral action behavior words contrast in the Body Project Treatment versus Interpersonal Psychotherapy condition‥

Analyses comparing BPT and IPT participants on change in neural response showed a significant condition × time interaction in the left mid cingulate cortex (MCC; MNI coordinates: −3, 2, 38, Z = 3.96, k = 34, Figure 2a; MNI coordinates: 0, 11, 41, Z = 3.84) in response to the thin model > average-weight model contrast and a significant condition × time interaction in the bilateral anterior cingulate cortex (ACC MNI coordinates: 6, 32, −7, Z = 4.99, k = 53, Figure 2b; MNI coordinates −3, 41, −7, Z = 4.79) in response to the eating disorder behavior words > neutral action behavior words contrast. BPT versus IPT participants showed a greater decrease in neural response in these regions. Pretest to posttest change in eating disorder symptoms did not significantly correlate with change in neural response in the mid cingulate cortex (*r* = −0.17) and anterior cingulate cortex (*r* = −0.11).

### Intervention effects for continuous and dichotomous outcomes

From pretest to posttest, BPT versus IPT participants showed significantly greater reductions in eating disorder symptoms (*d* = 0.54, *p* = 0.02) and body dissatisfaction (*d* = 0.57, *p* = 0.014) and marginally greater reductions in psychosocial impairment (*d* = 0.39, *p* = 0.088). Groups did not differ on change in pursuit of the thin ideal (*d* = 0.00, *p* = 0.893), anxiety symptoms (*d* = 0.34, *p* = 0.144), or depressive symptoms (*d* = 0.36, *p* = 0.114).

From pretest to 6-month follow-up, BPT versus IPT participants showed significantly greater decreases in body dissatisfaction (*d* = 0.68, *p* = .015) and psychosocial impairment (*d* = 0.63, *p* = 0.028), and marginally greater decreases in eating disorder symptoms (*d* = 0.53, *p* = 0.072). Groups did not differ on change in pursuit of the thin ideal (*d* = 0.37, *p* = 0.272), anxiety symptoms (*d* = 0.39, *p* = 0.246), or depressive symptoms (*d* = 0.33, *p* = 0.351).

At posttest (see Table 3), BPT versus IPT showed significantly higher abstinence from binge eating and purging (48% versus 23%, respectively; *d* = 0.68), but non-significantly different remittance from eating disorder diagnoses (48% vs 44%, respectively; *d* = 0.21). At 6-month follow-up, BPT versus IPT did not differ on abstinence from binge eating and purging (52% versus 54%, respectively; *d* = −0.17) and remittance from eating disorder diagnoses (82% vs 85%, respectively; *d* = −0.19).

**Table 3.** Logistic regression results for estimates of group differences in remission and abstinence rates at posttest and 6-month follow-up

Sample	Estimate	SE	*t*-value	*p*-value	OR	95% CI
Posttest						
Abstinence	1.23	0.616	3.97	0.046	3.42	1.02–11.43
Remission	0.55	0.495	1.22	0.270	1.73	0.66–4.55
Six-month follow-up						
Abstinence	−0.30	0.499	0.35	0.554	0.74	0.28–1.98
Remission	−0.12	0.636	0.04	0.843	0.88	0.25–3.06

*Note:* CI, confidence interval; OR, odds ratio; SE, standard error.

## Discussion

We tested whether BPT produced a greater reduction in eating disorder symptoms and brain reward region response to the thin ideal and behaviors used to pursue this ideal, as well as higher abstinence from eating disorder behaviors and remittance from eating disorder diagnoses than IPT. BPT produced significantly greater reductions in eating disorder symptoms than IPT through posttest, but this effect was only marginal by 6-month follow-up. The significant acute effect was a *d* = 0.54, a medium effect size, and was a *d* = .53 at follow-up. The within-condition eating disorder symptom reduction for BPT (*d* = 0.82) was similar to the average within-condition reduction (*d* = 1.05) from past trials that used other control conditions (Rohde et al., 2015; Stice et al., 2019; Stice et al., 2023; Stice, Rohde et al., 2023), suggesting that intervention effects are reproducible. Critically, these results replicate prior evidence that BPT produced significantly greater reductions in eating disorder symptoms than IPT (Stice et al., 2023). Also, as hypothesized, BPT produced significantly higher abstinence by posttest than IPT (48% versus 23%), though differences in abstinence were not significantly different by 6-month follow-up. However, differences in diagnostic remittance of eating disorders did not differ significantly across groups. We focus on abstinence and remittance at the end of treatment because past trials have reported these outcomes (Fairburn et al., 2015; Stice, Rohde, et al., 2023; Wonderlich et al., 2014). The 48% end of treatment abstinence rate was higher than the 23%–34% end of treatment abstinence rate produced by CBT-E, the 33% end of treatment abstinence rate produced by ICAT, and the 31% end of treatment abstinence rate produced by IPT (Fairburn et al., 2015; Stice, Rohde, et al., 2023; Wonderlich et al., 2014). Results suggest that not only is BPT much shorter than these alternative transdiagnostic treatments, but may also be more effective.

Although not all the hypothesized effects for the eating pathology outcomes reached significance in the current trial, this is one of only two transdiagnostic eating disorder treatments to produce significantly greater reduction in eating pathology than an alternative treatment; CBT-E likewise produced significantly larger reductions in global eating disorder pathology than IPT at posttest (Fairburn et al., 2015). Results suggest that reducing the valuation of the thin ideal and behaviors used to pursue this ideal is more effective in reducing eating disorder symptoms than improving interpersonal functioning.

BPT versus IPT participants showed significantly greater reductions in body dissatisfaction at posttest (*d* = 0.57) and 6-month follow-up (*d* = 0.68), significantly greater reductions in psychosocial impairment at 6-month follow-up (*d* = 0.63), and marginally greater reductions in psychosocial impairment (*d* = 0.39) at posttest. The significant effects were medium to large. These effects generally accord with findings from prior trials (Rohde et al., 2015; Stice et al., 2019; Stice et al., 2023; Stice, Rohde et al., 2023). We report marginal effects because we were only powered to detect medium-to-large effects. One important exception is that in the present trial, BPT did not produce greater reductions in pursuit of the thin-ideal, which we observed in prior trials that compared BPT to IPT (Stice, Rohde et al., 2023), usual care (Stice, Rohde, Butryn, et al., 2015), and waitlist controls (Stice et al., 2023). The mean reduction in pursuing the thin ideal was larger for IPT in the present trial versus the past trial, and the mean reduction in pursuing the thin ideal was smaller for BPT in the present trial versus past trials. Possibly, some participants in IPT groups in the present trial spontaneously discussed the negative effects of the thin ideal, even though this was not encouraged, which resulted in greater reductions in pursuing the thin ideal for IPT participants. BPT did not produce greater reductions in anxiety or depressive symptoms than IPT in the present trial, though it did produce greater reductions in anxiety symptoms than IPT previously (Stice, Rohde et al., 2023).

Regarding target engagement, BPT versus IPT participants showed a significantly greater reduction in MCC response to thin models and in ACC response to eating disorder behavior words. The MCC is a key region involved in multiple higher-order functions, including motor control, cognitive flexibility, conflict monitoring, and affective processing (Vogt, 2016). In the context of eating disorders, heightened MCC activation has been associated with increased emotional salience and self-referential processing of body image cues (Monteleone et al., 2018). The reduction in MCC activity among BPT participants may reflect attenuation of emotional reactivity to the thin ideal. Alternatively, given the MCC’s role in conflict monitoring and control, the reduction in MCC activity in BPT participants may also reflect a shift in attentional processing or cognitive disengagement from the thin ideal. The ACC is part of the salience network and the emotional and cognitive processing networks (Morris et al., 2022; Rolls, 2023). The ACC has been associated with the evaluation of action-outcome contingencies, conflict monitoring, and error detection (Rolls, 2023). The observed decrease in ACC response to eating disorder behavior words in BPT participants may indicate a reduced salience or emotional reactivity to eating disorder behavior cues, potentially due to a diminished reinforcement of these behaviors or internal drive associated with these behaviors. Alternatively, heightened ACC activity has been linked to increased sensitivity to self-relevant, often negative, emotional information and self-criticism (Dixon et al., 2022). Thus, the reduction in ACC activity in BPT participants may also reflect improvements in self-referential processing. In this context, the neural reduction in ACC activity in BPT participants could represent a broader reduction in internal distress or cognitive-emotional reactivity related to eating disorder behaviors.

It is important to acknowledge that our past target engagement trial found that BPT participants showed greater reductions in reward (caudate) and attention (precuneus) response to thin models compared to waitlist controls (Stice et al., 2023), but we did not observe reductions in these regions in response to thin models relative to IPT in the present trial. One potential explanation for this is that expectancies contributed to the reductions in pursuing the thin ideal for participants who completed an active treatment (IPT), which did not emerge in waitlist controls in the past trial. Another potential explanation for the limited replication is that only 69 participants provided complete fMRI data, versus 103 participants who provided complete fMRI data previously (Stice et al., 2023).

It is important to consider study limitations. First, the sample size was moderate, which limited sensitivity to detecting small effects and the ability to examine effects for specific eating disorder categories. Second, the 6-month follow-up period is relatively short.

In conclusion, the present study adds important confirmatory data to the BPT evidence base. Results indicate that the dissonance-based transdiagnostic BPT achieved strong engagement, high rates of behavioral abstinence and disorder remittance, large reductions in eating disorder symptoms, and produced objective target engagement support for reduced valuation of thinness and eating disorder behaviors, but not for high-calorie binge foods relative to transdiagnostic IPT. Its most marked effects were for reduced body dissatisfaction, but contrary to past trials, no effects were found for reductions in pursuit of the thin ideal.

Given that group-delivered treatments appear to be more cost-effective than treatments delivered on an individual basis, it may be useful to adapt other eating disorder treatments for group delivery. Further, dissemination efforts will be needed to motivate clinicians to learn and provide more cost-effective treatments. Broadly implementing shorter, more cost-effective transdiagnostic treatments may improve access to care for individuals with eating disorders, as cost is a key access barrier for individuals with these conditions.

This trial was registered at ClinicalTrials.gov (NCT03261050) and was supported by NIH grant MH111782. We thank Gary Glover for assisting with the paradigm development and fMRI data acquisition at the Lucus Center for Imaging. Correspondence should be addressed to Eric Stice, Dept of Psychiatry and Behavioral Sciences, 401 Quarry Road, Palo Alto, CA 94305.

## Supporting information

Stice et al. supplementary material Stice et al. supplementary material

## Data Availability

This is the first and only manuscript to describe the results from this trial. These findings have not been presented at any conferences.

## Supplementary material

The supplementary material for this article can be found at http://doi.org/10.1017/S0033291725102341.
