# Supplementary material for: Randomized target engagement trial of a dissonance-based transdiagnostic eating disorder treatment versus transdiagnostic interpersonal psychotherapy
Source: Psychol Med. 2025 Nov 6;55:e337. doi: 10.1017/S0033291725102341 (PMC13058642; doi:10.1017/S0033291725102341)
Supplement: Stice et al. supplementary material [file S0033291725102341sup001.docx]

Running Head: DISSONANCE-BASED EATING DISORDER TREATMENT

**Randomized Target Engagement Trial of a Dissonance-Based Transdiagnostic Eating Disorder Treatment versus Transdiagnostic Interpersonal Psychotherapy**

Eric Stice

Stanford University

Sonja Yokum

Paul Rohde

Oregon Research Institute

Cara Bohon

Heather Shaw

Stanford University

**Supplementary Methods**

**fMRI data acquisition**

Functional scans used a T2* weighted EPI plus sequence (72 slices, TE = 25 ms, TR = 2000 ms, flip angle = 90°, matrix size = 100 x 100, voxel size = 2 mm^3^, axial slices = 72, FOV = 200, multiband acceleration factor = 3). Structural scans were collected using a high-resolution anatomical T1-weighted MP-RAGE scan (TE = 3.43 ms, TR = 2500 ms, 256 x 256 matrix, voxel size = 1 mm^3^, sagittal slices = 176, FOV = 256). Upon arrival for their scan, participants reported the last time they ate.

**fMRI data preprocessing**

Neuroimaging data were preprocessed and analyzed using the Statistical Parametric Mapping (SPM12; Wellcome Department of Cognitive Neurology; [http://www.fil.ion. ucl.ac.uk/spm](http://www.fil.ion.ucl.ac.uk/spm)) in Matlab (Mathworks, Inc., Natick, MA). Anatomical images were skullstripped using the Brain Extraction Tool in the FMRIB Software Library (FSL; FMRIB Analysis Group, Oxford, UK). Anatomical images were segmented and normalized to Montreal Neurological Institute (MNI) space with the use of the Diffeomorphic Anatomical Registration Through Exponentiated Lie Algebra (DARTEL) toolbox, coregistered to the mean functional image, and segmented into six tissue types using unified segmentation approach (Ashburner & Friston, 2005). Functional data were adjusted for variation in magnetic field distortion using field maps, realigned to the mean functional scan from that run, coregistered with the anatomical, normalized to MNI space using the DARTEL template and deformation fields output, and smoothed to 6 mm Gaussian full-width-at-half-maximum. We used the Artifact Detection Toolbox (ART; Gabrieli Laboratory, McGovern Institute for Brain Research, Cambridge MA) to detect spikes in global mean response and motion outliers. Head motion ≥ 3 mm or degrees in any direction was our *a priori* exclusion criteria. Motion parameters < 3 mm were included as regressors in the design matrix at individual fixed effect analysis. Image volumes where the z-normalized global brain activation showed >1.5 mm of composite (linear plus rotational) movement were flagged as outliers and de-weighted during individual-level model estimation.

Complete fMRI data were available for 70 participants for the high-calorie binge food paradigm and eating disorder behavior word paradigm (BPT n = 37; IPT n = 33) and for 69 participants for the thin model paradigm (BPT n = 37; IPT n = 32).

**Supplemental References**

Ashburner, J., & Friston, K.J. (2005). Unified segmentation. *Neuroimage*. 26:839-851.
